# A fractional order epidemic model for the simulation of outbreaks of Ebola

**DOI:** 10.1186/s13662-021-03272-5

**Published:** 2021-03-10

**Authors:** Weiqiu Pan, Tianzeng Li, Safdar Ali

**Affiliations:** 1 School of Mathematics and Statistics, Sichuan University of Science and Engineering, Zigong, 643000 China; 2 South Sichuan Center for Applied Mathematics, Yibin, 644000 China

**Keywords:** Epidemic models, Fractional order model, Ebola, Modified grid approximation method

## Abstract

The Ebola outbreak in 2014 caused many infections and deaths. Some literature works have proposed some models to study Ebola virus, such as SIR, SIS, SEIR, etc. It is proved that the fractional order model can describe epidemic dynamics better than the integer order model. In this paper, we propose a fractional order Ebola system and analyze the nonnegative solution, the basic reproduction number $R_{0}$, and the stabilities of equilibrium points for the system firstly. In many studies, the numerical solutions of some models cannot fit very well with the real data. Thus, to show the dynamics of the Ebola epidemic, the Gorenflo–Mainardi–Moretti–Paradisi scheme (GMMP) is taken to get the numerical solution of the SEIR fractional order Ebola system and the modified grid approximation method (MGAM) is used to acquire the parameters of the SEIR fractional order Ebola system. We consider that the GMMP method may lead to absurd numerical solutions, so its stability and convergence are given. Then, the new fractional orders, parameters, and the root-mean-square relative error $g(U^{*})=0.4146$ are obtained. With the new fractional orders and parameters, the numerical solution of the SEIR fractional order Ebola system is closer to the real data than those models in other literature works. Meanwhile, we find that most of the fractional order Ebola systems have the same order. Hence, the fractional order Ebola system with different orders using the Caputo derivatives is also studied. We also adopt the MGAM algorithm to obtain the new orders, parameters, and the root-mean-square relative error which is $g(U^{*})=0.2744$. With the new parameters and orders, the fractional order Ebola systems with different orders fit very well with the real data.

## Introduction

Ebola is very dangerous and fatal. After its existence was found in 1976 in the Ebola River region of southern Sudan and Congo (old Zaire), it caused quite a stir in the medical community. After this, people call this infectious disease “Ebola”. The Ebola virus can break the internal organs of the human body and once infected with this virus, people’s blood will flow out, with a lethal rate of between 50% and 90%. On July 17, 2019, the World Health Organization (WHO) proclaimed that the Ebola virus outbreak in the African country Congo (DRC) should attract global attention. On June 1, 2020, a new round of Ebola outbreak broke out in Northwest of Congo. This is the 11th outbreak of Ebola in Congo (DRC) since 1976.

At present, the total deaths caused by the Ebola virus in the world is approximately 12,999 [1]. In the latest outbreak, Guinea, Liberia, and Sierra Leone, the three countries with the most severe epidemics, have claimed 14,000 lives. In addition to the three foreign countries in West Africa, there were eight deaths in Nigeria, two deaths in Mali, and one death in the United States due to Ebola infection. As a result, it is necessary to study the spread of infection. After extensive search, at present, it is a mystery what is the reservoir of the Ebola virus. Many experts believe that the reservoir includes fruit bats [2]. Once you are exposed to the body fluids, secretions, tissues, and many more, you may be infected with Ebola virus and have some symptoms within 2–21 days. During 4–10 days, a person has the ability to spread pathogen. When people are infected with Ebola virus, symptoms such as headache, general fever, stomach discomfort leading to vomiting and bloody diarrhea will appear. Therefore, some diseases like malaria and typhoid are often misdiagnosed as Ebola. There are also some mathematical models to study Ebola virus. Rachah et al. [3] applied the optimization theory to research the impact of vaccination on Ebola virus which spread among people. In [4], Althaus et al. used the SEIR model to elaborate the development trend of the EBOV epidemic, and the numerical solution obtained by the predictive correction method is very close to the actual number of infections reported in Guinea, Sierra Leone, and Liberia. Rachah et al. [5] explained the diffuse of Ebola virus on the basis of the SEIR model and how to control Ebola virus in the most effective way. Ndanguza et al. [6] used the Markov chain Monte Carlo algorithm to study the 1995 epidemic in the Democratic Republic of Congo using the number of symptomatic infections and deaths, demonstrating that the infection rate of Ebola virus is 99.95% and the mortality rate is 98.6%.

The calculus invented by Newton and Leibniz plays a critical role in modern mathematics and classical mathematics. Fractional calculus is a theory with respect to differentiation and integration of any order and is a generalization of integer order calculus. In the past three centuries, researchers mainly studied the fractional calculus in the pure theoretical field of mathematics. However, with the development of modern engineering, we can see the application of fractional order differential equations in various fields [7–9]. Then, more and more scholars at home or abroad concentrate on exploring the theory of fractional calculus. Now, there are a lot of scientific fields that involve the fractional order differential equations. Both the theoretical analysis and numerical calculation of the fractional order differential equations are particularly urgent. Because fractional calculus has a memory function, this function ensures the influence of historical information on the past and the future. Therefore, when studying the fractional order models, fractional calculus can achieve our ideal results [10]. There are many kinds of viruses in the world. Combining the fractional calculus with biological infectious disease models can allow us to more accurately understand the development of the epidemic. Then Area et al. [11] discussed the fractional order Ebola system with the same order in terms of the Riemann–Liouville fractional order derivative. Tulu [12] put forward a fractional order Ebola model in terms of the Caputo fractional order derivative to simulate the number of deaths caused by Ebola virus. González-Parra [13] explained and understood influenza A (H1N1) with a nonlinear fractional order model. Ariel [14] explored the Leptospira outbreak pattern from the spread of Leptospirosis in the population and from animals to humans. Other than that, there are some other models, such as coronavirus [15–18], the anthrax disease [19], human liver [20], and so on. In many literatures, most of the models are normal with the same order, and the parameters of the Ebola model are either existing or predicted. There are two aspects that need to be improved: one is improving the fitting effect between the numerical solution of these systems and the real data; the other is that different orders can be considered.

In this article, we mainly study the fractional order Ebola system in terms of Caputo fractional order derivative. Firstly, we research the fractional order Ebola system with the same order, then a fractional order Ebola system with different orders is proposed. The GMMP scheme [21] is the method that we take to get the numerical solution of the SEIR fractional order Ebola system. This will make the calculation cost low. There are some other numerical methods, such as q-homotopy analysis transform method (q-HATM) [15, 19, 20, 22], the fractional Euler method (FEM) [23], discretized collocation method [24], variational iteration method(VIM) [16], fractional natural decomposition method (FNDM) [17], and so on. Then we adopt the MGAM method [25] to estimate the parameters of literature [11]. With the new fractional orders and parameters, the numerical solution of the SEIR fractional order Ebola system is closer to the real data than the models in other literature sources.

The composition of this article is as follows. In Sect. 2, three classical fractional derivatives are introduced, and the expression of fractional order Ebola system is shown. In Sect. 3, some properties of the fractional order Ebola model are introduced, including the nonnegative solution, the basic reproduction number $R_{0}$, and the stabilities of equilibrium points. In Sect. 4, we introduce the GMMP method, including the stability and convergence. In Sect. 5, we show the MGAM method for parameter estimation. In Sect. 6, the numerical simulation of the SEIR fractional order Ebola model is studied and compared with real data. In Sect. 7, we have a sum up for the paper.

## Fractional derivatives and the SEIR fractional order Ebola systems

### The three most commonly used fractional derivatives

Compared to the integer order calculus, the fractional order calculus has more advantages. For example, fractional order calculus has a memory function and can catch the entire properties of the function. Fractional calculus is a better description of the dynamical behavior of a system. So it has a great influence on the development of scientific research [26]. The most influential definitions for fractional order derivatives include Riemann–Liouville (R-L), Caputo, and Grünwald–Letnikov (G-L) definition [27]. In 1847, the German mathematician Riemann made further additions on this basis and formed the first more complete definition of fractional calculus, i.e., the Riemann–Liouville definition

1 $$\begin{aligned}& {}^{RL}_{a}D_{t}^{\alpha }f(t)= \frac{1}{\Gamma (n-\alpha )} \frac{d^{n}}{dt^{n}} \int _{a}^{t}(t-\nu )^{n-\alpha -1}f(\nu )\,d\nu , \end{aligned}$$

where $n-1<\alpha <n$, $n\in Z^{+}$, and $\Gamma (z)=\int _{0}^{\infty } t^{z-1}e^{-t}\,dt$ is the gamma function.

The R-L definition has a high position in theoretical analysis. However, Caputo derivative with initial value conditions is more suitable for modern engineering applications, especially in viscoelastic theory and solid fluid mechanics. The expression of Caputo derivative definition is explained in the following way:

2 $$\begin{aligned}& {}^{C}_{a}D_{t}^{\alpha }f(t)= \frac{1}{\Gamma (n-\alpha )} \int _{a}^{t}(t- \nu )^{n-\alpha -1}f^{(n)}( \nu )\,d\nu , \end{aligned}$$

where $n-1<\alpha <n$, $n\in Z^{+}$.

For the purpose of getting numerical solutions of the fractional order differential equations, we introduce the concept of Grünwald–Letnikov fractional order derivative. The definition of Grünwald–Letnikov fractional order derivative is given as follows:

3 $$\begin{aligned} {}^{GL}_{a}D_{t}^{\alpha }f(t)&=\lim _{\substack{h\rightarrow 0\\mh=t}}h^{- \alpha }\sum_{r=0}^{m}(-1)^{r} \binom{\alpha }{r} f(t-rh) \\ &=\sum_{k=0}^{n} \frac{f^{(k)}(\alpha )(t-\alpha )^{k-\alpha }}{\Gamma (k+1-\alpha )}+ \frac{1}{\Gamma (m+1-\alpha )}\cdot \int _{a}^{t}(t-\nu )^{m-\alpha }f^{(m+1)}( \nu )\,d\nu , \end{aligned}$$

where $n-1<\alpha <n$ and $f(t)$ has n-order continuous derivative on the interval $[a,t]$.

By the definitions of fractional order derivative, we can obtain that the G-L fractional derivative and the R-L derivative are equivalent, while the Caputo derivative and the R-L derivative are not. Their difference can be shown as follows:

4 $$\begin{aligned}& {}^{RL}_{a}D_{t}^{\alpha }f(t)={{}^{C}_{0}D_{t}^{\alpha }}f(t)+ \sum_{k=0}^{n-1}r_{k}^{ \alpha }(t)f^{(k)}(a), \end{aligned}$$

where $n-1<\alpha <n$, $n\in Z^{+}$, and $f^{(k)}(a)$, ($k=0,1,\ldots ,n-1$) are the initial conditions. We also consider $r_{k}^{\alpha }=\frac{t^{k-\alpha }}{\Gamma (k+1-\alpha )}$.

In this paper, we prefer the Caputo operator and concentrate on the case $n=1$, namely $\alpha \in (0,1)$. Thus, equation (4) becomes

5 $$\begin{aligned}& {}^{RL}_{a}D_{t}^{\alpha }f(t)={{}^{C}_{0}D_{t}^{\alpha }}f(t)+r_{0}^{ \alpha }f(a), \end{aligned}$$

where $0<\alpha <1$ and $f(a)$ is the initial condition. We also consider $r_{0}^{\alpha }=\frac{t^{-\alpha }}{\Gamma (1-\alpha )}$.

### The SEIR fractional order Ebola system

Firstly, the integer order model of Ebola epidemic is presented. According to the characteristics of infectious disease models, it is better to divide the total human population *N* into four sub-populations: $S(t)$ susceptible humans, $E(t)$ exposed humans, $I(t)$ infection humans, $R(t)$ removed humans. Using [11], the Ebola classical differential equation is as follows:

6 $$\begin{aligned}& \frac{dS(t)}{dt}=-\frac{\beta S(t)(qE(t)+I(t))}{N}, \\& \frac{dE(t)}{dt}=\frac{\beta S(t)(qE(t)+I(t))}{N}-\delta E(t), \\& \frac{dI(t)}{dt}=\delta E(t)-\gamma I(t), \\& \frac{dR(t)}{dt}=\gamma I(t), \end{aligned}$$

where $q\in [0,1]$ is an adjustable factor. The parameters denote different meanings respectively:

(i) *γ* is the recovery rate;
(ii) *δ* is the per-capita infectious rate;
(iii) $\beta =pc$, where *p* is the probability that a healthy person will be infected with Ebola when exposed to an infected person, and *c* is the per-capita contact rate. Hence, *β* is an average number of people infected after touching with an infected person who has already developed symptoms. It varies every day.

The parameters have been acquired from [11]. They are

7 $$ \gamma =\frac{1}{7}, \quad\quad \delta = \frac{1}{12}. $$

And the initial conditions are as follows:

8 $$ S(0)=AM*\frac{m}{100},\quad\quad E(0)=0, \quad\quad I(0)=15, \quad\quad R(0)=0. $$

Among them $AM=18{,}805{,}278$ is the total population of these countries, including Guinea, Liberia, and Sierra Leone, and *m* is the number to be determined which is the proportion of susceptible population to total population. So far, there has been no empirical evidence that humans are completely immune to viruses. We can set $50\leq m<100$, $R(0)>0$. It is a reasonable assumption.

**Figure 1:**
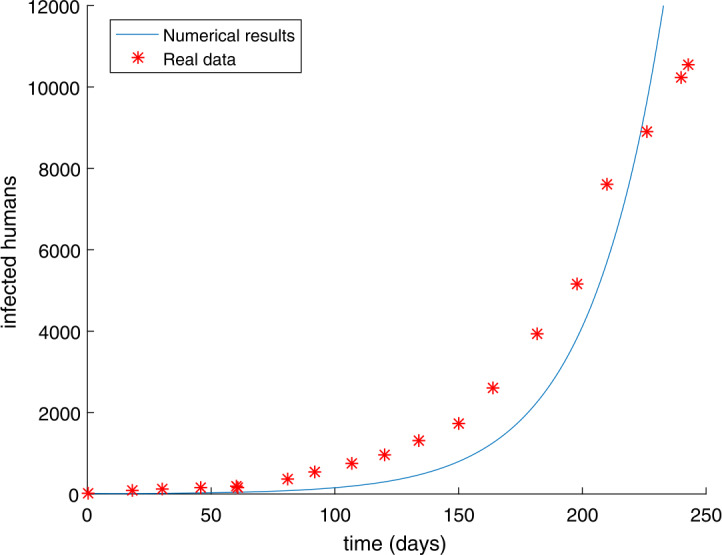
The number of Ebola infections $I(t)$ in Guinea, Liberia, and Sierra Leone compared with the numerical results of classical model (6) obtained by ODE45 function. The values of the parameters are $\beta =0.2305$, $m=80$, $q=0.0300$ and the root-mean-square relative error is $g(U)=1.0061$

The initial conditions indicate that the number of people infected was small at the beginning. If preventive measures were taken promptly at that time, then the number of next infections would be less. We can use the ODE45 function in Matlab to solve the solution of nonlinear differential equation (6). Table 1 reveals the number of Ebola infections in these countries, including Guinea, Liberia, and Sierra Leone from March 27 to December 1, 2014. We get the simulation result shown in Fig. 1, in which the root-mean-square relative error is $g(U)=1.0061$, demonstrating that the effect of match could be improved further to reflect the spread of Ebola. The fractional calculus has more physical significance than the integer order calculus, the most important of which is that the fractional order derivative offers good ideas for describing the memory and genetic characteristics of different materials. Recently, many fractional order infectious disease systems have been proposed [15, 16, 19, 28]. Hence, the fractional order Ebola system with the same order is depicted as follows:

9 $$\begin{aligned}& \lambda _{\alpha }{}_{0}^{C}D_{t}^{\alpha }S(t)=- \frac{\beta S(t)(qE(t)+I(t))}{N}, \\& \lambda _{\alpha }{}_{0}^{C}D_{t}^{\alpha }E(t)= \frac{\beta S(t)(qE(t)+I(t))}{N}-\delta E(t), \\& \lambda _{\alpha }{}_{0}^{C}D_{t}^{\alpha }I(t)= \delta E(t)-\gamma I(t), \\& \lambda _{\alpha }{}_{0}^{C}D_{t}^{\alpha }R(t)= \gamma I(t), \end{aligned}$$

where ${}_{0}^{C}D_{t}^{\alpha }$ means the Caputo fractional order derivative with $\alpha \in (0,1)$.

**Table 1:** These data are statistics on the number of people who have tested positive for Ebola in Guinea, Liberia, and Sierra Leone, which is a cumulative process

Date	Guin	Lib	S. Leone	Total
27/03/2014	15	0	0	15
14/04/2014	71	0	0	71
26/04/2014	121	0	0	121
12/05/2014	138	6	0	144
27/05/2014	163	6	7	176
16/06/2014	254	18	92	364
02/07/2014	292	54	211	557
17/07/2014	301	76	368	745
30/07/2014	337	109	507	953
13/08/2014	376	190	733	1299
29/08/2014	482	322	935	1739
12/09/2014	678	654	1287	2619
01/10/2014	950	927	2076	3953
17/10/2014	1217	965	2977	5159
29/10/2014	1391	2515	3700	7606
14/11/2014	1647	2562	4683	8892
17/10/2014	1217	965	2977	5159
17/10/2014	1217	965	2977	5159
28/11/2014	1892	2753	5595	10,240
01/12/2014	1921	2801	5831	10,553

Here, the expression of parameters *β*, *γ*, *δ*, and *q* has been explained in classical model (6) and *N* is the total population. However, we can see that units on the left of system (9) are not same as those on the right. To be specific, they are going to be the dimensions of $(\mathrm{days})^{-\alpha }$ and the dimensions of $(\mathrm{days})^{-1}$. If we take the left-hand side of this system (9) by $\lambda _{\alpha }$ which has the dimension of $(\mathrm{days})^{\alpha -1}$, then we can have the same units $(\mathrm{days})^{-1}$ on both sides of this system. Generally speaking, we can get $\lambda _{\alpha }=1$ in the fractional order system. In this paper, we use the MGAM methods to find a suitable set of fractional orders and parameters that make the fractional order Ebola system to provide numerical results that agree well with the real data.

## The properties of the model

In this chapter, we analyze the basic properties and the stabilities of fractional order model (9). The first thing to note is that when the initial values are nonnegative, the solutions of model (9) are always nonnegative. Then, we present the basic reproduction number $R_{0}$ and provide the condition for the stabilities of the disease-free equilibrium (DFE).

### The nonnegative solution of the model

#### Lemma 1

([10])

*The Laplace transform of the Caputo fractional derivative* ${}^{C}_{a}D_{t}^{\alpha }f(t)$ *has the following form*:

10 $$\begin{aligned}& L \bigl\{ {}^{C}_{a}D_{t}^{\alpha }f(t);s \bigr\} =s^{\alpha }F(s)-\sum_{k=0}^{n-1}s^{ \alpha -k-1}f^{(k)}(0), \end{aligned}$$

*where* $n-1\leq \alpha < n$, $F(s)= L\{f(t);s\}=\int _{0}^{+\infty }e^{-st}f(t)\,dt$.

#### Lemma 2

([29]; Generalized mean value theorem)

*Assume that* $f(t)\in C[a,b]$ *and* ${}^{C}_{a}D_{t}^{\alpha }f(t)\in C[a,b]$ *for* $0<\alpha \leq 1$, *then we obtain*

11 $$\begin{aligned}& f(t)=f(a)+\frac{1}{\Gamma (\alpha +1)}{{}^{C}_{a}}D_{\eta }^{\alpha }f( \eta ) (t-a)^{\alpha }, \end{aligned}$$

*where* $a\leq \eta \leq t$ *for all* $t\in (a,b]$.

It follows from Lemma 2 that if ${}^{C}_{a}D_{t_{0}}^{\alpha }f(t_{0})>0$, $t_{0}\in (a,b)$, there is a neighborhood *D* of $t_{0}$ such that $f(t)>f(a)$, $\forall t\in D$. If ${}^{C}_{a}D_{t_{0}}^{\alpha }f(t_{0})<0$, $t_{0}\in (a,b)$, there is a neighborhood *D* of $t_{0}$ such that $f(t)< f(a)$, $\forall t\in D$.

#### Theorem 1

*The region* $\Omega _{+}={(S,E,I,R);S\geq 0,E\geq 0,I\geq 0,R\geq 0}$ *is a positive invariant of system* (9).

#### Proof

From literature [30], the existence and uniqueness of the solution of system (9) on the time interval $(0,+\infty )$ can be obtained. On the hyper-planes of region $\Omega _{+}$, we have

12 $$\begin{aligned}& {}^{C}_{0}D_{t}^{\alpha }S | _{S=0}=0, \end{aligned}$$

13 $$\begin{aligned}& {}^{C}_{0}D_{t}^{\alpha }E | _{E=0}= \frac{\beta S(t)I(t)}{N}, \end{aligned}$$

14 $$\begin{aligned}& {}^{C}_{0}D_{t}^{\alpha }I | _{I=0}= \delta E(t), \end{aligned}$$

15 $$\begin{aligned}& {}^{C}_{0}D_{t}^{\alpha }R | _{R=0}= \gamma I(t). \end{aligned}$$

We apply the Laplace transform to formula (10)

$$ s^{\alpha }F(S)-s^{\alpha -1}S(0)=0. $$

Thus, if $S(0)$ belongs to the hyper-plane $S=0$, then $S(t)=0$, $t>0$. Therefore, the hyper-plane $S=0$ is a positive invariant set. If $(S(0),E(0),I(0),R(0))\in \Omega _{+}$, according to Eqs. (12)–(15) and Lemma 2, the solution $S(t)$, $E(t)$, $I(t)$, $R(t)$ cannot escape from the hyper-planes of $E=0$, $I=0$, $R=0$; i.e., the region $\Omega _{+}$ is a positive invariant set. □

### The basic reproduction number $R_{0}$

According to system (9), we can easily get the disease-free equilibrium (DFE) as $E_{0}=(N,0,0,0)$. The next-generation operator approach [31] is adopted to calculate $R_{0}$. We first need to obtain the Jacobian matrices *F* (the new infection terms) and the Jacobian matrices *V* (the remaining transfer term):

16 $$ F= \begin{pmatrix} \beta q & \beta \\ \delta & 0 \end{pmatrix},\quad\quad V= \begin{pmatrix} \delta & 0 \\ 0 &\gamma \end{pmatrix}. $$

$V^{-1}$ is as follows:

17 $$ V^{-1}= \begin{pmatrix} \frac{1}{\delta } & 0 \\ 0 & \frac{1}{\gamma } \end{pmatrix}. $$

Thus,

18 $$ FV^{-1}= \begin{pmatrix} \frac{\beta q}{\delta } & \frac{\beta }{\gamma } \\ 1 & 0 \end{pmatrix}. $$

The calculation method of the basic reproduction number is as follows:

$$\begin{aligned}& R_{0}=\rho \bigl(FV^{-1} \bigr), \end{aligned}$$

where *ρ* denotes the spectral radius of a matrix $FV^{-1}$. Therefore, we have

19 $$\begin{aligned}& R_{0}= \frac{\beta q +\sqrt{\beta ^{2}q^{2}+4\frac{\beta \delta ^{2}}{\gamma }}}{2\delta }. \end{aligned}$$

### The stabilities of equilibrium points

The Jacobian matrix evaluated at DFE is

20 $$ J(E_{0})= \begin{pmatrix} A& 0 \\ B &0 \end{pmatrix}, $$

where the matrices *A* and *B* are

21 $$ A= \begin{pmatrix} 0& C \\ 0&F-V \end{pmatrix},\quad\quad B= \begin{pmatrix} 0& 0&\gamma \end{pmatrix}, $$

with the matrices *C* and $F-V$ being given by

22 $$ C= \begin{pmatrix} -\beta q& -\beta \end{pmatrix}, \quad\quad F-V= \begin{pmatrix} \beta q-\delta & \beta \\ \delta & -\gamma \end{pmatrix}. $$

Notice that the eigenvalues of the matrix $J(E_{0})$ consist of zero and $\lambda ^{*}$, where $\lambda ^{*}$ represents the eigenvalues of matrix $F-V$. The meaning of the matrix *F* is the new infection terms and the matrix *V* is the remaining transfer term. For convenience, let the matrix $F-V$ be the matrix *D*. The local stability of DFE is assessed by the eigenvalues of the characteristic equation $\operatorname{det} (\lambda ^{*} I-D)$ which is

23 $$\begin{aligned}& \bigl(\lambda ^{*} \bigr)^{2}+a\lambda ^{*}+b=0, \end{aligned}$$

where $a=\gamma +\delta -\beta q$, $b=\delta \gamma -\gamma \beta q-\delta \beta $.

This is a second order polynomial. Applying the Routh–Hurwitz criteria [32], if all coefficients of characteristic equation (23) are positive, the maximum real part of all the eigenvalues of the matrix *D* are negative. Therefore, we give the following theorem.

#### Theorem 2

*The disease*-*free equilibrium* $E_{0}$ *of fractional order system* (9) *is locally stable if* $R_{0}<1$ *and the coefficient* *a* *of characteristic equation* (23) *satisfies* $a>0$. *If* $R_{0}>1$, *then* $E_{0}$ *is unstable*.

#### Proof

According to the expression of $R_{0}$, when $R_{0}<1$, we can obtain $\delta \gamma >\gamma \beta q+\beta \delta $. Thus, the coefficient *b* of the characteristic equation (23) is positive. And, from the condition $a>0$. Based on the Routh–Hurwitz criteria, the maximum real part of all the eigenvalues of the matrix *D* is negative. It is worth noting that the eigenvalues of the matrix $J(E_{0})$ involve zero. In response to this situation, literature [33] has already discussed. We can obtain that the disease-free equilibrium $E_{0}$ of fractional order system (9) is locally stable. On the contrary, if $R_{0}>1$, based on the Routh–Hurwitz criteria, the coefficient *b* must be negative. There is a positive number in the maximum real part of the eigenvalues of the matrix $J(E_{0})$. Thus, the disease-free equilibrium $E_{0}$ of fractional order system (9) is not stable. □

Next, we will research the global asymptotic stability at the disease-free equilibrium.

#### Theorem 3

*The disease*-*free equilibrium* $E_{0}$ *of fractional order system* (9) *is globally asymptotically stable if* $R_{0}<1$ *and the coefficient* *a* *of characteristic equation* (23) *satisfies* $a>0$.

#### Proof

Since $S< N$ for fractional order system (9), for the second equation of system (9), we have

24 $$\begin{aligned}& {}^{C}_{0}D_{t}^{\alpha }E(t)< \beta qE(t)+\beta I(t)-\delta E(t). \end{aligned}$$

For comparison, define a linear system given by (24) with equality, namely

25 $$\begin{aligned}& {}^{C}_{0}D_{t}^{\alpha }E(t)= \beta qE(t)+\beta I(t)-\delta E(t). \end{aligned}$$

Now, we observe the following system:

26 $$\begin{aligned}& {}^{C}_{0}D_{t}^{\alpha }E(t)=\beta qE(t)+ \beta I(t)-\delta E(t), \\& {}^{C}_{0}D_{t}^{\alpha }I(t)=\delta E(t)- \gamma I(t). \end{aligned}$$

It has the coefficient matrix $F-V$. Through the discussion of Theorem 2, system (26) satisfies $\lim_{t\rightarrow \infty }E=0$ and $\lim_{t\rightarrow \infty }I=0$. With the help of comparison theorem [34], and noting (24), it follows that these limits also hold for the second and third equations of fractional order system (9). For the remaining equations of system (9), we have $\lim_{t\rightarrow \infty }R=0$ and $\lim_{t\rightarrow \infty }S=N$. Thus, for $R_{0}<1$ and the coefficient *a* of characteristic equation (23) satisfying $a>0$, the disease-free equilibrium $E_{0}=(N,0,0,0)$ is globally asymptotically stable. □

#### Remark 1

Because the disease-free equilibrium and the endemic equilibrium point of fractional order system (9) are the same, the stabilities analysis to the endemic equilibrium point can refer to the process of disease-free equilibrium.

## Numerical method for the fractional order differential equation

### Description of the GMMP method

Simply, system (9) can be expressed as the form of $\lambda \odot {}_{a}^{C}D_{t}^{\alpha }y(t)=f(t,y(t))$, where $y(t)=(S(t),E(t),I(t),R(t))^{T}$. We consider a uniform grid in $[a,t]$, $a=t_{0}< t_{1}< t_{2}<\cdots <t_{N}=t$, $t_{i+1}-t_{i}=\Delta t=h$. Meanwhile, $y(t)$ is assumed to be continuous in each finite interval $(a,t)$ with $t < T$.

Based on the classical notation of finite differences

27 $$ \frac{1}{h^{\alpha }}\Delta _{h}^{\alpha }y(t)= \frac{1}{h^{\alpha }} \Biggl(y(x_{n})- \sum_{k=0}^{n}c_{k}^{\alpha }y(x_{n-k}) \Biggr), $$

where $c_{k}^{\alpha }=(-1)^{k}\binom{\alpha }{k}$ is the binomial coefficient and $\alpha \in (0,1)$.

Then, both Riemann–Liouville and Grünwald–Letnikov fractional order derivatives could be rewritten as the following formula:

28 $$ {}_{a}^{RL}D_{t}^{\alpha }y({t})={}_{a}^{GL}D_{t}^{\alpha }y({t})= \lim_{h \rightarrow 0}\frac{1}{h^{\alpha }}\Delta _{h}^{\alpha }y(t) \approx \frac{1}{h^{\alpha }}\Delta _{h}^{\alpha }y(t). $$

It is worth noting that the difference term $r_{0}^{\alpha }$ between the Caputo and Riemann–Liouville fractional order derivative (5) also works on the uniform grid. Thus, the Caputo fractional order derivative can be expressed as follows:

29 $$ {}_{a}^{C}D_{t}^{\alpha }y(t) \approx \frac{1}{h^{\alpha }} \Biggl(y(x_{n})-\sum _{k=0}^{n}c_{k}^{\alpha }y(x_{n-k}) \Biggr)-r_{n}^{ \alpha }y(a), $$

where $c_{k}^{\alpha }=(-1)^{k}\binom{\alpha }{k}$ is the binomial coefficient, $y(a)$ is the initial condition, and $r_{n}^{\alpha }=r_{0}^{\alpha }(t_{n})=\omega _{0,-1}^{\alpha }(n)^{- \alpha }$. The meaning of the function *ω* is $\omega _{\mu ,\nu }^{\alpha }= \frac{\Gamma (\mu \alpha +1)}{\Gamma (\nu \alpha +1)}$, $\mu ,\nu \in \mathbb{N}_{0}\cup \{-1\}$. And $r_{n}^{\alpha }y(a)$ tends to zero when $n\rightarrow \infty $.

The first step is to obtain the difference grid according to the solution area, and the continuous solution domain of the differential equation is expressed as a finite number of grids; the second step is to obtain the difference quotient by calculation, which is used to replace the differential term in the differential equation; the third step is in the discrete case getting the difference equation, which contains a finite number of unknown variables. This is the basic process of the Gorenflo–Mainardi–Moretti–Paradisi scheme (GMMP). You can see [21, 35, 36] for details. In general, the fractional order nonlinear equation can be presented using Caputo operator

30 $$\begin{aligned}& \lambda \odot {}_{a}^{C}D_{t}^{\alpha }y(t)=f \bigl(t,y(t) \bigr),\quad 0\leq t \leq T, \\& y^{(k)}(a)=y_{0}^{(k)},\quad k=0,1,\ldots ,n-1, \end{aligned}$$

where ${}_{a}^{C}D_{t}^{\alpha }$ represents the Caputo operator.

We combine (29) with (30) to get the formula that

31 $$\begin{aligned} y(x_{n})=h^{\alpha }\oslash \lambda \odot f \bigl(t_{n},y(x_{n}) \bigr)+\sum_{k=0}^{n}c_{k}^{ \alpha }y(x_{n-k})+h^{\alpha }r_{n}^{\alpha }y_{0}. \end{aligned}$$

Through the above simple reasoning, the solution form (31) of the fractional order nonlinear equation has been obtained. Any Caputo fractional order differential equation with initial value problem can be numerically solved by formula (31). We need to bring in the specific equations and use the Matlab software (R2016a) to solve them. It is worth mentioning that the code we used was written by ourselves. Since there is an unknown variable $y(x_{n})$ on both sides of (31), we choose the Newton algorithm to gain the value of $y(x_{n})$ via equation (31).

### Stability of the GMMP method

If the GMMP method is not stable, it may lead to absurd numerical solutions. Therefore, it is necessary to study the stability of the GMMP method. To present the stability of the GMMP method, we consider the following fractional differential equation:

32 $$\begin{aligned} {}_{a}^{C}D_{t}^{\alpha }y(t)=f \bigl(t,y(t) \bigr)=my(t), \end{aligned}$$

where $m>0$ is the coefficient and $y(0)=y_{0}$ is the initial condition. Applying equation (31), we obtain

33 $$\begin{aligned} y(x_{n})= \bigl(1- \bigl(h^{\alpha }\oslash \lambda \bigr) m \bigr)^{-1} \Biggl\{ \sum_{k=0}^{n}c_{k}^{ \alpha }y(x_{n-k})+h^{\alpha }r_{n}^{\alpha }y_{0} \Biggr\} . \end{aligned}$$

The *λ* is used to make sure that both sides of this equation have the same dimension which is explained in fractional order system (9). Generally speaking, we can get $\lambda =1$. Thus, equation (33) becomes

34 $$\begin{aligned} y(x_{n})= \bigl(1-mh^{\alpha } \bigr)^{-1} \Biggl\{ \sum_{k=0}^{n}c_{k}^{\alpha }y(x_{n-k})+h^{ \alpha }r_{n}^{\alpha }y_{0} \Biggr\} . \end{aligned}$$

#### Lemma 3

([37])

*Assume that* $\{\xi _{n}\}$, $\{\rho _{n}\}$, *and* $\{\eta _{n}\}$ *are nonnegative sequences and*

35 $$\begin{aligned} \xi _{n}=\rho _{n}+\sum _{i=0}^{n-1}\eta _{i}\xi _{i} \quad \textit{for } n\geq 0. \end{aligned}$$

*Then it holds that*

36 $$\begin{aligned} \xi _{n}=\rho _{n}+\sum _{i=0}^{n-1}\rho _{i}\eta _{i} \prod_{j=i+1}^{n-1}(1+ \eta _{j}) \quad \textit{for } n\geq 0. \end{aligned}$$

#### Lemma 4

([37])

*Assume that* $\alpha \in (0,1)$ *for all the coefficients* $c_{k}^{\alpha }>0$ *defined in* (27) *shows the properties*

37 $$\begin{aligned} 0< c_{n}^{\alpha }< \cdots < c_{k}^{\alpha }< \cdots < c_{1}^{\alpha }< c_{0}^{ \alpha }=\alpha \quad \textit{and} \quad c_{k}^{\alpha }=O \biggl( \frac{1}{k^{1+\alpha }} \biggr) \quad (k\rightarrow \infty ). \end{aligned}$$

*What is more*, *the coefficients* $r_{n}^{\alpha }$ *and the new coefficients explained by* $S_{\mu ,n}^{\alpha }=\sum_{k=0}^{n}c_{k}^{\alpha }(n-k)^{\mu \alpha }$ *have the following properties when* $n\geq 0$:

38 $$\begin{aligned}& \alpha =S_{0,0}^{\alpha }< S_{0,1}^{\alpha }< \cdots < S_{0,n-1}^{\alpha }< S_{0,n}^{ \alpha }< 1, \\& 0< r_{n}^{\alpha }< r_{n-1}^{\alpha }< \cdots < r_{1}^{\alpha }< r_{0}^{ \alpha }= \frac{1}{\Gamma (1-\alpha )}< 1. \end{aligned}$$

#### Theorem 4

*The numerical solutions* (31) *obtained by the MGAM method are absolute stable if the condition* $\vert (1-mh^{\alpha })^{-1}\leq 1 \vert $ *is satisfied*.

#### Proof

Because $c_{k}^{\alpha }$ and $r_{n}^{\alpha }$ in (34) are positive and the initial value $y_{0}$ can be negative or positive, the values $y(x_{n})$ can be replaced by their absolute values $\vert y(x_{n}) \vert $. Combining the condition $\vert (1-mh^{\alpha })^{-1}\leq 1 \vert $, equation (34) becomes

39 $$\begin{aligned} \bigl\vert y(x_{n}) \bigr\vert \leq \sum _{k=0}^{n}c_{k}^{\alpha } \bigl\vert y(x_{n-k}) \bigr\vert +h^{\alpha }r_{n}^{ \alpha } \vert y_{0} \vert . \end{aligned}$$

Let $\xi _{n}= \vert y(x_{n}) \vert $, $\rho _{n}=r_{n}^{\alpha } \vert y_{0} \vert $, $\eta _{n}=c_{k}^{\alpha }$. Based on Lemma 3, the following result is gained:

40 $$\begin{aligned} \bigl\vert y(x_{n}) \bigr\vert \leq \sum _{k=0}^{n}r_{k}^{\alpha }c_{n-k}^{\alpha } \prod_{j=k+1}^{n} \bigl(1+c_{n-j}^{ \alpha } \bigr)+r_{n}^{\alpha } \vert y_{0} \vert . \end{aligned}$$

It follows from Lemma 4 that $r_{k}^{\alpha }<1$ and $0< c_{k}^{\alpha }\leq S_{0,n}^{\alpha }<1$. Thus, the product in (40) is deduced by

41 $$\begin{aligned} \prod_{j=k+1}^{n} \bigl(1+c_{n-j}^{\alpha } \bigr)\leq \prod _{j=0}^{n} \bigl(1+c_{n-j}^{ \alpha } \bigr)\leq \exp \bigl(S_{0,n}^{\alpha } \bigr)< \exp (1), \end{aligned}$$

and the sum is deduced by

42 $$\begin{aligned} \sum_{k=0}^{n}r_{k}^{\alpha }c_{n-k}^{\alpha } \leq S_{0,n}^{\alpha }< 1. \end{aligned}$$

Thus, it follows from equations (41) and (42) that

43 $$\begin{aligned} \bigl\vert y(x_{n}) \bigr\vert < \exp (1)+r_{n}^{\alpha } \vert y_{0} \vert . \end{aligned}$$

As you can see, there exists a constant *K* such that $\exp (1)+r_{n}^{\alpha } \vert y_{0} \vert \leq K$ for all $n\geq 0$. Equation (43) can be rewritten as

44 $$\begin{aligned} \bigl\vert y(x_{n}) \bigr\vert \leq K. \end{aligned}$$

Thus, the numerical solutions (31) obtained by MGAM method are absolute stable. □

### Convergence of the GMMP method

To present the convergence of the GMMP method, we also consider specific function (32). We define the global error $\vert e_{n} \vert $ between the true value $y_{n}$ and the approximate value $y(x_{n})$ as

45 $$\begin{aligned} \vert e_{n} \vert = \vert q_{n}+p_{n} \vert , \end{aligned}$$

where $q_{n}=y_{n}-y(\tilde{x}_{n})$ and $p_{n}=y(\tilde{x}_{n})-y(x_{n})$ with $y(\tilde{x}_{n})=(1-mh^{\alpha })^{-1} \{\sum_{k=0}^{n}c_{k}^{\alpha }y_{n-k}+h^{ \alpha }r_{n}^{\alpha }y_{0}\}$.

#### Theorem 5

([37])

*The numerical solutions* (31) *obtained by the MGAM method are convergent when the global error* $\vert e_{n} \vert $ *satisfies*

46 $$\begin{aligned} \vert e_{n} \vert \leq C\sum _{k=0}^{n-1}c_{k}^{\alpha } \vert q_{n-k} \vert + \vert p_{n} \vert , \end{aligned}$$

*where* $C\leq (1-mh^{\alpha })^{-1}\exp ((1-mh^{\alpha })^{-1})$.

#### Proof

It follows from [37, Theorem 6.2] that we obtain

47 $$\begin{aligned} \vert p_{n} \vert \leq \bigl(1-mh^{\alpha } \bigr)^{-1}\sum_{k=0}^{n-1}c_{k}^{\alpha } \vert e_{n-k} \vert . \end{aligned}$$

Combining with (45), the following result is valid:

48 $$\begin{aligned} \vert e_{n} \vert \leq \bigl(1-mh^{\alpha } \bigr)^{-1}\sum_{k=0}^{n-1}c_{k}^{\alpha } \vert e_{n-k} \vert + \vert q_{n} \vert . \end{aligned}$$

Using Lemma 3, (46) holds. From Lemma 4, we can get $c_{k}^{\alpha }=O(h^{1+\alpha })$ for $h\rightarrow 0$, $n\rightarrow \infty $. And according to [37, Corollary 6.1], $q_{n}=O(h^{1+\alpha })$, $h\rightarrow 0$ is deduced. Thus, for equation (46), the convergence order is expected to be one. □

## Parameter estimation method for fractional order nonlinear dynamic system

The reported Ebola cases are those who tested positive for Ebola virus by a laboratory. Now, the testing methods recommended by the WHO are as follows: automatic or semi-automatic nucleic acid testing for routine diagnostic management is adopted. If nucleic acid testing is not possible in remote areas, rapid antigen testing can be used. It is recommended to use rapid antigen detection methods during screening as part of monitoring activities, but reactivity testing should be confirmed by nucleic acid testing.

In order for the numerical solution of the fractional order model to be closer to the real number of infected people, we must estimate and correct the original parameters. At this time, a fractional order dynamical system could be depicted as the following form with uncertain parameters:

49 $$\begin{aligned}& \lambda \odot {}_{a}^{C}D_{t}^{\alpha }y(t)=f \bigl(t,h(t) \bigr),\quad 0\leq t \leq T, \\& y^{(k)}(a)=y_{0}^{(k)},\quad k=0,1,\ldots ,n-1, \end{aligned}$$

where $y=(y_{1},y_{2},\ldots ,y_{n})^{T}$ and $f=(f_{1},f_{2},\ldots ,f_{n})^{T}$ are *n*-dimensional vector functions, and $h_{i}$ ($i=1,2,\ldots ,n$) represents uncertain parameters $u_{i}$ ($i=1,2,\ldots ,p$), *p* is the number of parameters.

In literature [11], the author used the predictor-corrector PECE method of Adams–Bashforth–Moulton to solve numerically the fractional order differential equations. In order to make the numerical solutions of fractional order differential equations fit well with the real data, the author should find the best value of the parameter *q* that minimizes the $L_{2}$ norm between the real data and the model, defined by

$$ \xi =\sqrt{\sum_{j=1}^{a} \vert c_{j}-x_{j} \vert ^{2}}, $$

where $c_{j} $ is the cumulative number of infected people according to the data at day *j* and $c_{j}$ is the prediction proposed by the model. If the value of *ξ* is smaller, it means that the number of infections predicted by this model is closer to the real data. However, the author still did not explain how to find the optimal value *q*, and there will be a big error in judging the results of fitting with real data based on the value of *ξ*.

Next, in this paper, we need to find the optimal parameters to make the numerical solution of the fractional order Ebola system as close as possible to the number of people infected with Ebola adopting MGAM algorithm. This process is very complicated and requires strict calculations, and we use Matlab (R2016a) to write code to realize it. The steps are as follows:

(i) For certain parameters, the GMMP method can be adopted to get the numerical solution $y(t_{j})$ of fractional order differential equation (9). This result cannot be used as our final data. Next, we look for the optimal result.

(ii) *D* is composed of many closed intervals as Cartesian products and it is bounded. So there is no doubt that $(u_{1}, u_{2}, \ldots , u_{m})\in D$.

50 $$ D= \bigl[u_{1}^{(\min )}, u_{1}^{(\max )} \bigr]\times \bigl[u_{2}^{(\min )}, u_{2}^{(\max )} \bigr] \times \cdots \times \bigl[u_{m}^{(\min )}, u_{m}^{(\max )} \bigr]. $$

Take step length $h_{j}$ to refine the interval $[u_{j}^{(\min )}, u_{j}^{(\max )}]$, ($j=1,2,\ldots ,m$), and get

51 $$ G(D)= \bigl\{ U\in D: u_{i,j}=u_{i}^{(\min )}+k_{i} \times h_{i}, i=1,2,\ldots ,m, j=0,1,\ldots ,M_{j} \bigr\} . $$

This means that we need to find the optimal parameters $U=(u_{1}, u_{2}, \ldots , u_{m})$ in $G(D)$.

(iii) Calculate the root-mean-square relative error function

52 $$ g \bigl(U^{*} \bigr)=\min_{U\in G(D)} \biggl\{ \sqrt{ \frac{\sum_{j=0}^{N}((y(t_{j})-x_{j})/x_{j})^{2}}{N+1}} \biggr\} , $$

where $y(t_{j})$ is the numerical solution of fractional order system equation (33) for given parameters $U=(u_{1},u_{2},\ldots ,u_{m})$, and $x_{j}$ is the actual data. Then we can estimate the precise parameters $U^{*}$ within the given domain $G(D)$. In this paper, we selected the number of people infected with Ebola at 20 time points. So, we can choose *N* as 20.

(iv) If the step $h_{j}$ is inappropriate, it will be difficult for us to find the optimal result in the grid $G(D)$. Here is a method, when you do not get better $g(U^{*})$ under the first set step, you need to adjust the step $h_{j}$ to get another $g(U^{**})$. Next, as long as they satisfy $\Vert g(U^{**}) \Vert <\delta $ or $\Vert U^{*}-U^{**} \Vert <\varepsilon $, where *δ* and *ε* are small error parameters, this proves we find an approximate estimate of the parameter vector which is $U^{**}$. Otherwise, let $U^{*}=U^{**}$, turn to the first step (i).

## Fitting the numerical solution of the fractional order Ebola model with real data

Unlike the classical differential equation theory, solving the fractional order differential equations is very difficult. The solutions obtained by the classical method are only approximate solutions. There are many methods to solve the fractional order differential equations, such as the Mellin transform method, power series method, and Babenko’s symbolic calculus method [10]. Therefore, in this section, we use the GMMP method to give the numerical results and the MGAM method is used to acquire the parameters for the SEIR fractional order Ebola model. With the new parameters and orders, the results demonstrate that it matches closer with the real data than the SEIR integer-order Ebola system (6). From this, a conclusion can be gained which is the new parameters and orders are more precise. Meanwhile, the fractional order Ebola system with different orders (53) using the Caputo fractional order derivative is studied. We also use the MGAM algorithm to gain new orders and parameters. With the new parameters and orders, the fractional order Ebola system with different orders fits very well with the real data.

### SEIR fractional Ebola epidemic model with the same orders

From Sect. 5, we should be aware that the parameters that need to be estimated are the Ebola transmissibility *β*, the fractional order *α*, *q*, and *m*. The unknown parameter vector is written as $U=(\alpha ,\beta ,q,m)$. The choice of parameter intervals to narrow the target value is crucial for the result. In light of the reality, the intervals and the step $h_{j}$ are chosen in the following way:

$$\begin{aligned}& 0\leq \alpha =u_{1}\leq 1, \quad\quad 0\leq \beta =u_{2} \leq 1, \quad\quad 0\leq q=u_{3}\leq 1, \quad\quad 50\leq m=u_{4}< 100, \end{aligned}$$

and

$$\begin{aligned}& h_{1}=h_{2}=h_{3}=h_{4}=0.01. \end{aligned}$$

**Figure 2:**
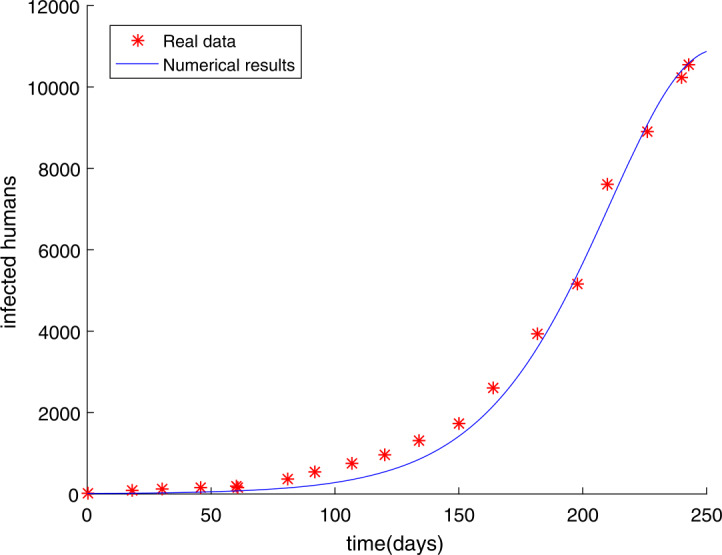
The number of Ebola infections $I(t)$ in Guinea, Liberia, and Sierra Leone compared with the numerical results of fractional order system (9) obtained by the MGAM method and $g(U^{*})=0.4146$

**Figure 3:**
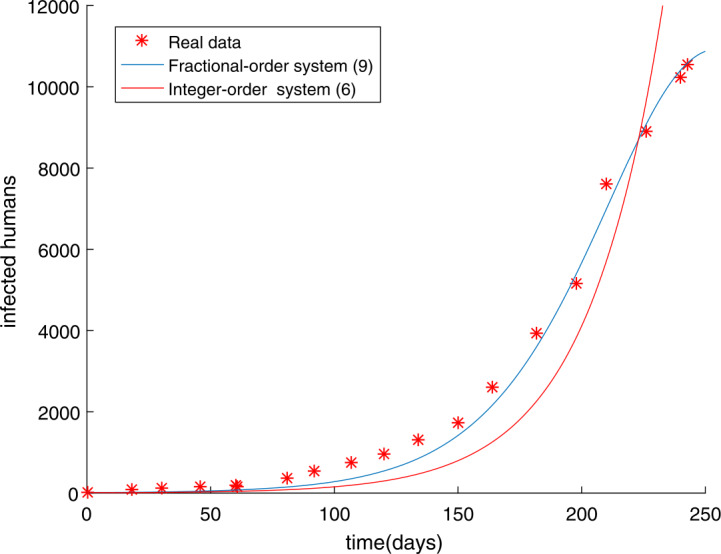
The fitting results of SEIR integer-order Ebola system (6) in which the root-mean-square relative error is $g(U)=1.0061$ and fractional-order Ebola system (9) in which the root-mean-square relative error is $g(U^{*})=0.4146$ with real data

**Figure 4:**
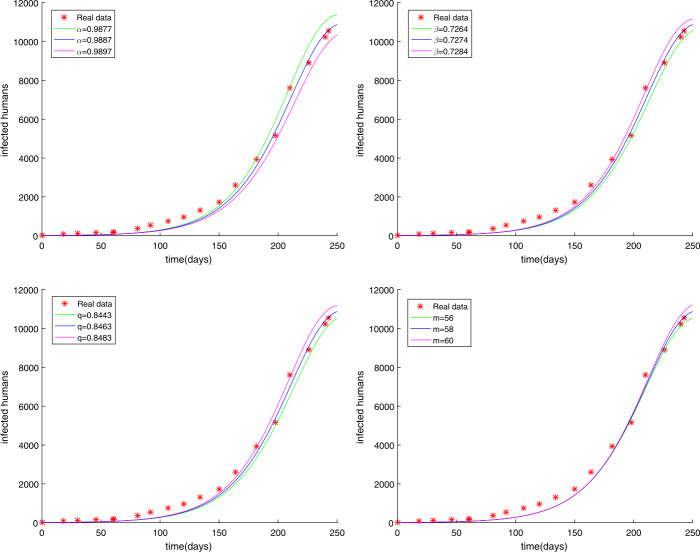
The influence of *α*, *β*, *q*, *m* on the number of Ebola infections $I(t)$, when the remaining parameters are fixed

The initial value has not been changed and the time is chosen as $t = 250$ days. In Sect. 4, we have used the modified GMMP method to obtain the numerical solution and use the parameter estimation method again in Sect. 5. We acquire the results $U^{*}$ as follows:

$$\begin{aligned}& \alpha =u_{1}=0.9887, \quad\quad \beta =u_{2}=0.7274, \quad\quad q=u_{3}=0.8463, \quad\quad m=u_{4}=58, \end{aligned}$$

with $g(U^{*})=0.4146$. The simulation results in Fig. 2 show that the parameter value $U^{*}$ obtained by using the MGAM method makes the fractional order Ebola system fit very well with the real data compared with Fig. 1. We compare Fig. 1 with Fig. 2 to prove that our method is effective, as shown in Fig. 3. By observing how the parameters influence the variety of infected people $I(t)$ when other parameters are unchanged, we can draw the conclusion that the parameter measured by the MGAM method is the ideal parameter. The results show that the four parameters have an impact on the number of infected people $I(t)$. The influence of orders and parameters is reflected in Fig. 4, which shows that our estimated parameters are indeed the ideal parameters.

### SEIR fractional order Ebola epidemic model with different orders

Many fractional order epidemic models have the same order [38, 39]. Hence, the fractional order Ebola system with different orders using Caputo derivatives is studied as follows:

53 $$\begin{aligned}& \lambda _{\alpha _{1}}{}_{0}^{C}D_{t}^{\alpha _{1}}S(t)=- \frac{\beta S(t)(qE(t)+I(t))}{N}, \\& \lambda _{\alpha _{2}}{}_{0}^{C}D_{t}^{\alpha _{2}}E(t)= \frac{\beta S(t)(qE(t)+I(t))}{N}-\delta E(t), \\ & \lambda _{\alpha _{3}}{}_{0}^{C}D_{t}^{\alpha _{3}}I(t)= \delta E(t)- \gamma I(t), \\ & \lambda _{\alpha _{4}}{}_{0}^{C}D_{t}^{\alpha _{4}}R(t)= \gamma I(t), \end{aligned}$$

where *β*, *γ*, *δ*, *q* are the same as in (6), *N* is the total population, and $\alpha \in (0,1)$. We have introduced the parameter $\lambda _{\alpha _{i}}$ ($i=1,2,3,4$) to ensure that the dimensions of both sides of fractional order equations (53) are equal. For convenience, we have taken $\lambda _{\alpha _{i}}=1$ ($i=1,2,3,4$) in fractional order system (53). From Sect. 5, we should be aware that the parameters that need to be estimated are $\alpha _{1}$, $\alpha _{2}$, $\alpha _{3}$, $\alpha _{4}$, *β*, *q*, *m*. The unknown parameter vector is written as $U=(\alpha _{1},\alpha _{2},\alpha _{3},\alpha _{4},\beta ,q,m)$. The choice of parameter intervals to narrow the target value is crucial for the result. In light of the reality, the intervals and the step $h_{j}$ are chosen in the following way:

$$\begin{aligned}& 0\leq \alpha _{1}=u_{1}\leq 1 , \quad\quad 0\leq \alpha _{2}=u_{2}\leq 1 , \quad\quad 0\leq \alpha _{3}=u_{3}\leq 1 , \quad\quad 0\leq \alpha _{4}=u_{4}\leq 1 , \\ & 0\leq \beta =u_{5}\leq 1 , \quad\quad 0\leq q=u_{6}\leq 1 , \quad\quad 50\leq m=u_{7}< 100 , \end{aligned}$$

and

$$\begin{aligned}& h_{1}=h_{2}=h_{3}=h_{4}=h_{5}=h_{6}=h_{7}=0.01. \end{aligned}$$

**Figure 5:**
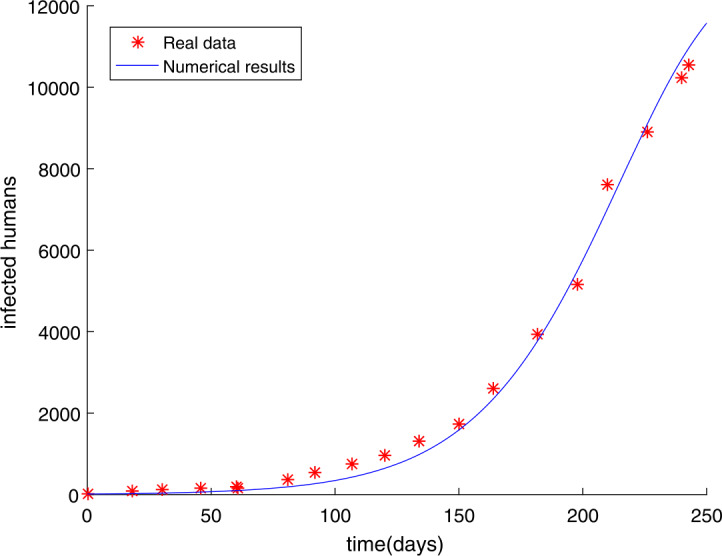
The number of Ebola infections $I(t)$ in Guinea, Liberia, and Sierra Leone compared with the numerical results of fractional order system (53) obtained by the MGAM method and $g(U^{*})=0.2744$

**Figure 6:**
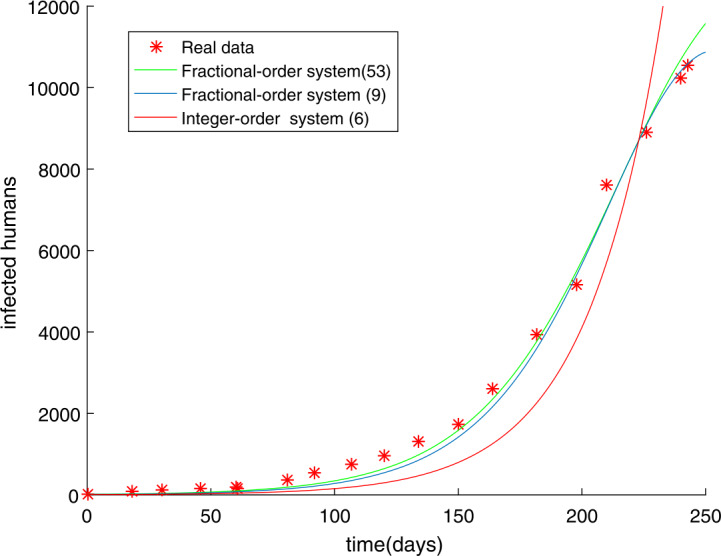
The fitting results of integer order system (6), fractional order Ebola system (9) which has the same order, and fractional order Ebola system (53) which has different orders with the real data. Their root-mean-square relative errors are $g(U)=1.0061$, $g(U^{*})=0.4146$, and $g(U^{*})=0.2744$ respectively

**Figure 7:**
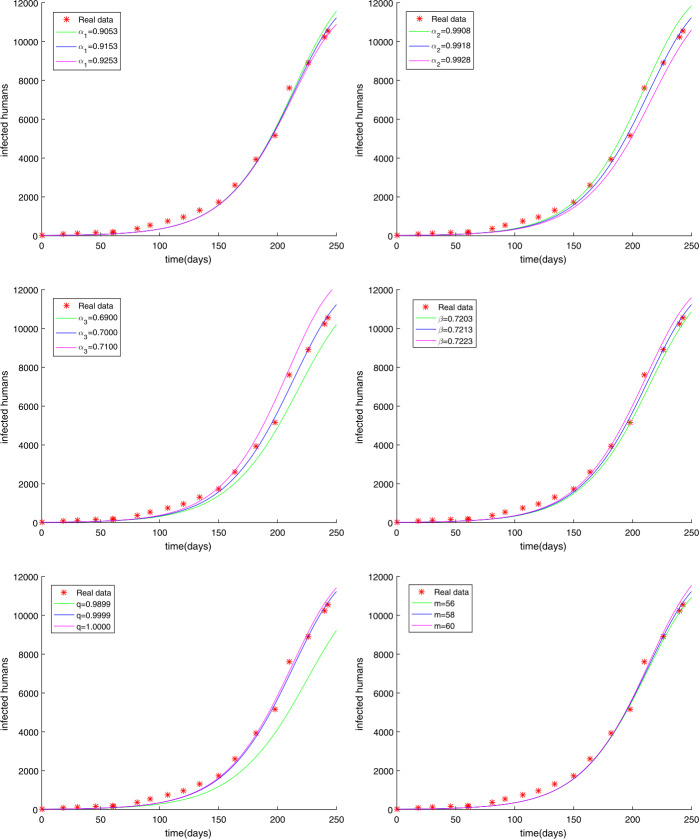
The influence of $\alpha _{1}$, $\alpha _{2}$, $\alpha _{3}$, *β*, *q*, *m* on the number of Ebola infections $I(t)$, when the remaining parameters are fixed

We get the results $U^{*}$ as follows:

$$\begin{aligned}& \alpha _{1}=u_{1}=0.9153 , \quad\quad \alpha _{2}=u_{2}=0.9918 , \quad\quad \alpha _{3}=u_{3}=0.7000 , \quad\quad \alpha _{4}=u_{4}=0.9567 , \\ & \beta =u_{5}=0.7213 , \quad\quad q=u_{6}=0.9999 , \quad \quad m=u_{7}=58 , \end{aligned}$$

with $g(U^{*})=0.2744$. The simulation results show that the parameter value $U^{*}$ obtained by using the MGAM method makes the fractional order Ebola system fit very well with the real data, which is shown in Fig. 5. To prove that the fractional order Ebola model of different orders (53) is better than that of the same order (9), we made a comparison, see Fig. 6. By observing how the parameters influence the variety of infected people $I(t)$ when other parameters are unchanged, we can obtain the conclusion that the method is very good and the parameter we estimated is indeed the ideal parameter. The results show that when $\alpha _{4}$ changed, the number of infected people $I(t)$ basically did not change, while other parameters had a certain impact on $I(t)$. The influence of orders and parameters is reflected in Fig. 7.

## Conclusion

In this paper, we researched fractional order Ebola mathematical models with the same order or different orders. The nonnegative solution, the basic reproduction number $R_{0}$, and the stabilities of equilibrium points for the SEIR fractional order Ebola system are analyzed. Considering the numerical solution acquired from the GMMP scheme, which is stable and convergent, we use the MGAM method to estimate the parameters of the SEIR fractional order Ebola system. With the new fractional orders and parameters, Fig. 2 and Fig. 5 show that the numerical solutions fit well with the real data, which proves that the GMMP scheme and the MGAM are efficient and valid for parameter estimation. The real data begins at the Ebola infection that broke out in the countries of Guinea, Liberia, and Sierra Leone in 2014. By comparing the fitting effects illustrated in Fig. 3 and Fig. 6 of integer order Ebola model (6) and fractional order Ebola models (9), (53) with the real data, we can conclude that we have indeed made improvements on the basis of literature [11]. Figure 4 and Fig. 7 show the effect of every parameter on the number of infected humans $I(t)$ with the other parameters fixed. In addition, from the root-mean-square relative error $g(U^{*})=0.4146$ of fractional order model (9), in which the order is the same, and the root-mean-square relative error $g(U^{*})=0.2744$ of fractional order model (53), in which the orders are different, we get the conclusion that the fractional order Ebola model with different orders can provide a better fitting with the real data than other models. However, there is still some work that needs to be done. Next, we will improve this model to satisfy a type of infectious disease.

## Data Availability

The data used to support the findings of this study are available from the corresponding author upon request.
